# Common oral conditions and correlates: an oral health survey in Kwara State Nigeria

**DOI:** 10.1186/s13104-017-2894-0

**Published:** 2017-11-07

**Authors:** Abiola O. Tobin, Ikeoluwapo O. Ajayi

**Affiliations:** 1 Sobi Specialist Hospital, Ministry of Health, P. O. Box 5871, Ilorin, Kwara State Nigeria; 2 Department of Epidemiology and Medical Statistics, Faculty of Public Health, College of Medicine University of Ibadan, Ibadan, Nigeria

**Keywords:** Oral conditions, Decayed missing filled teeth index, Prevalence, Socio-demographic factors

## Abstract

**Background:**

Oral diseases are one of the most prevalent health problems today with distribution and severity varying in different parts of the world and within the same country. Oral health surveys are needed to determine prevalence of oral conditions and the nature and urgency of oral health interventions. A modified version of World Health Organisation pathfinder survey methods was used to determine prevalence of oral conditions amongst 150 respondents in two local government areas in Kwara State, Nigeria. This involved a stratified cluster sampling technique which identified the subgroups; location and certain age groups 5–6, 12 and 35–44 years age groups respectively. Clinical oral examination was carried out to determine the presence and types of common oral conditions among the respondents. Data analysis was done using descriptive statistics and Chi square analysis at 5% level of significance.

**Results:**

Among all the selected subjects 91.3% had an oral condition, while for the rural and urban population it was 93.3 and 89.3% respectively (p > 0.05). The most prevalent oral conditions were plaque and surface calculus found in 66.0% of the respondents respectively. Others are gingivitis (30.0%), enamel wear (15.0%) and dental caries (13.0%). The mean decayed missing filled teeth index was 0.26. The decayed missing filled teeth index did not show any significant difference between the rural and urban areas or male and female gender. The presence of calculus (p = 0.005) and gingivitis (0.015) was more in males than females. The presence of plaque (0.001) and calculus (0.006) was significantly more among the skilled workforce. The 12 year age group had significantly more cases of plaque, calculus and gingivitis while there were more cases of enamel wear among the 35–44 year olds compared with other age groups. There were more cases of trauma (87.5%) seen in urban than rural location (p = 0.029).

**Conclusion:**

Oral health in selected communities of Kwara State is suboptimal requiring intervention. The presence of oral conditions is influenced by age, occupation, location and gender.

**Electronic supplementary material:**

The online version of this article (10.1186/s13104-017-2894-0) contains supplementary material, which is available to authorized users.

## Background

Oral diseases are one of the most prevalent health problems in the world today [1]. However, because the most commonly known of these diseases which are dental caries and periodontal diseases do not have a high mortality, their public health impact has not been appreciated [2]. The distribution and severity of oral diseases vary in different parts of the world and within the same country or region [3]. Globally the burden of oral disease has shown that dental caries still affects 60–80% of schoolchildren and majority of adults in industrialized countries [4].

The severity of dental caries among 35–44 year olds is high with (decayed, missing filled teeth index) DMFT > 13.9 in parts of North America, S. America and Europe. It is moderate DMFT 9–13.9 in parts of Asia and South Africa. It is low DMFT 5–8.9 and very low DMFT < 5.0 in parts of Africa and Asia [4]. Dental caries prevalence in adolescents in Lagos, Nigeria was 17.8% in 2002 and 23.8% in 2006 [5]. For periodontal diseases the most severe form community periodontal score (CP4) is seen among 10–15% of the adult population, while the most prevalent type (CP2) is characterized by gingival bleeding and calculus and is primarily a reflection of poor oral hygiene [6]. Oral cancer is the eighth most common cancer worldwide, it’s more prevalent in males and it affects 1–10 cases per 100,000 in many countries [4]. In Nigeria 2.3% of cancer cases are oral cancer but most patients present late in hospitals for treatment [7]. The relationship of human immunodeficiency virus (HIV) and oral health has also been demonstrated in a study in Lagos University Teaching Hospital where 40–50% of patients with HIV had oral disease and 43% of them had oral candidiasis [8]. World wide data on dental trauma is mostly unavailable, in Latin America dental trauma is reported in 15% of schoolchildren and 5–12% in school children in the Middle East [4]. Data from industrialised countries show dental traumatic injuries are on the increase from 16 to 40% among 6 year olds and 4 to 33% among 12–14 year olds. This has been attributed to more road traffic accidents, unsafe playgrounds and violence [4]. A similar scenario has been seen in developing countries like Nigeria due to interpersonal violence as well as motor vehicle and motor cycle accidents [9].

Epidemiological surveys of oral health conditions provide information on distribution and severity of major oral diseases and conditions. This helps to provide information on the extent to which oral health services and programmes match the current need of care as well as the nature of required preventive, curative and restorative services [10]. There are other studies which have used epidemiological surveys to determine oral health conditions in their communities. These authors have used the World Health Organisation (WHO) pathfinder survey method to determine current levels of dental diseases as well as dental caries prevalence [11, 12].

In Nigeria the types of oral health services available vary with the widest range available at teaching hospitals which are located in the urban centres [13]. The cost of treatment is high at the government owned clinics and higher at the private dental clinics. Consequently the demand for oral health care is generally poor compared with the need of the community. Individuals generally present late with orofacial swellings, pain and other complications leaving tooth extraction as the only option [14]. It has been identified that the provision of oral health services in primary health settings in Kwara State has been affected by lack of staff trained on oral health. This paper describes the procedure used to carry out an oral health survey and identifies the prevalent oral health conditions and associated factors among selected communities in Kwara State. The knowledge and understanding of common oral diseases existing in these communities can assist in the development of appropriate interventions as well as a focused training curriculum relevant to the oral health needs of the communities. The data obtained from this study was a segment of a larger study to evaluate a training intervention on the provision of oral health services among primary health care workers in Kwara State, Nigeria in 2009.

## Methods

The study was carried out on several sites in Ilorin West (urban) and Irepodun (rural) local government areas (LGAs) of Kwara State.

The pathfinder method was used to determine prevalence of oral diseases at the community level [10]. This method is a stratified cluster sampling technique which aims to include the most important subgroups in a population likely to have different disease levels such as location, ethnic groups and so on. In this study the subgroups were the urban and rural locations. It also uses certain age groups which have been predetermined by WHO to provide information about the severity of oral disease for these age groups and their treatment needs. The length of time the target population resided in a location at the time of examination was not taken into consideration as it was not a requirement in the WHO pathfinder survey which this study was patterned after [10]. The age groups recommended by WHO are 5–6 years for primary teeth and 12, 15, 35–44 and 65–74 years for permanent teeth. In this study three age groups were chosen; 5–6, 12 and 35–44 years [10]. The proof of age considered for identification of the various age groups was the bio-data in the school or staff records, however the subjects also knew their ages. The sample size was not calculated, rather the number of subjects chosen in each age group according to the WHO survey method depends on whether the expected prevalence of oral disease is low or high. In areas where low prevalence is expected a total of 25 subjects with an approximate male:female ratio is sufficient. In each of the two local government areas representing the urban and rural locations a minimum sample of 25 per age group was therefore used. However the total sample size for each of the three age groups (5–6, 12, 35–44 years) was 50 for both Irepodun and Ilorin west LGA’s and an overall sample size of 150 used in the study [10]. The sampling technique used to obtain this number in each age group was random sampling.

### Procedure

#### 5–6 and 12 years

A list of schools were obtained from the relevant authority and selection done from amongst them by random sampling using balloting; subjects in 5–6 years age group were recruited from nursery/primary schools and 12 year olds from junior secondary schools in the LGAs. Muslim school Omu-aran (Irepodun LGA) and Hiwanu Nasirudeen Adabata universal basic education (UBE) school (Ilorin West LGA) were selected for 5–6 year olds. Fourteen males and 11 females each were selected from Irepodun LGA and Ilorin West respectively.

In the 12 year old category the selected schools were Aperin Comprehensive Community School Omu-aran in Irepodun LGA, Baboko Community junior secondary school Ilorin and Government Girls Day Secondary School Oko-erin Ilorin in Ilorin West LGA. Thirteen males and 12 females each were selected from Irepodun LGA and Ilorin West LGA respectively. A list of students in the age group were obtained from bio-data of the students in each school’s record. The corresponding classes to these ages were obtained with the help of the principal/headmaster and an assigned teacher, the students were stratified by sex. The sample size of 25 was selected by balloting to approximate an equal male–female ratio.

#### 35–44 years

Subjects in the 35–44 years age group were recruited from the neighbourhood and Girls Secondary School Oko-erin in Ilorin West LGA. In Irepodun LGA they were recruited from Muslim school Omu-aran, Aperan Community College and the local government secretariat Omu-aran. The schools were among those earlier selected by balloting in the 5–6 and 12 years age group and selection done where teachers met the age criteria. This was done with the assistance of the principal and local government staff. The staff records formed the sampling frame which was stratified by sex and was used to select the individuals corresponding to the age group 35–44 years.

The individuals from the neighbourhood were selected by selecting a particular street within the LGA starting from a roundabout. Every willing adult that met the age requirement within the length of the street was examined in households till the sample size of 25 for Ilorin West was attained. Ten males and 15 females were selected from Ilorin west and similarly from Irepodun LGA. The multiple sites arose from the need to get individuals that fell within the age group.

### Clinical examination

Two investigators carried out the oral examination; the principal investigator and another investigator who were both qualified dental surgeons. The principal investigator trained the second investigator on criteria for classification of the findings and appropriate recording according to the study objectives. Calibration was achieved by inter and intra examiner reproducibility. Each investigator first practiced on a group of about ten subjects then independently examined ten percent of the subjects and compared findings. These were repeated till a consistency of uniform measurement was achieved using a common standard. Also duplicate examinations of a percentage of the sample were repeated by the same examiner to remove intra examiner variability. The investigators were not blinded to the participants location as to whether urban or rural. The only samples excluded were those exceeding the required number in a particular age group or which did not have required socio demographic information. These were removed before analysis.

All subjects were examined seated on a chair under natural light by the principal and second investigator.

The examinations were conducted in line with the procedures outlined by the WHO using disposable mouth spatulas, disposable hand gloves and dental mirrors [10]. Simple visual examination to determine the presence of surface calculus and plaque was done. This was to give information on the presence or absence of either of these conditions and not the degree or progression of the condition. DMFT index which measures caries experiences of individuals and is a reflection of teeth lost or conserved was recorded. Diagnosis of dental caries was accepted for teeth with obvious cavitation. Early caries detectable only by probing was excluded. The DMFT (decayed, missing, filled teeth) for permanent dentition and dmft (decayed, missing, filled teeth) index for primary dentition as well as the treatment needs were recorded for each subject. The DMFT index was calculated by counting the number of teeth which are decayed, missing or filled for each individual. Subjects were also examined for the presence of periodontal conditions or diseases as well as the presence of tumours, dental abscess, cellulitis, other abnormal enlargements, precancerous lesions, oral manifestations indicative of HIV, cancrum oris, fractures, enamel wear and any other presenting condition.

### Statistical methods

Data was recorded using a modified version of the World Health Organisation (WHO) oral health record form. Analysis was performed using Statistical Package for Social Sciences (SPSS version 17.0). Descriptive and inferential statistical analysis was carried out. Descriptive statistics such as frequency tables and cross tabulations were used to summarize the data. The results of basic demographic data were expressed in percentages. Pearson Chi square was used to test association between oral conditions and occupation, gender and location. Statistical significance level was set at 0.05 (p value).

## Results

### Socio demographic characteristics of individuals examined

There were 50 persons for the two local government areas in each of the selected age groups thus 5–6 years (50), 12 years (50) and 35–44 years (50). The male gender was 74 (49%) while females were 76 (51%). Among the parents of respondents those engaged in skilled occupations were highest in the age groups 5–6 years [35 (70.0%)] and 12 years [19 (38.0%)]. In the 5–6 age group, unskilled, civil servants, teachers and other professions were about the same in number 4 (8.0%). In the 12 year age group, teachers and other professions were the next highest group 10 (20.0%) followed by the civil servants 8 (16.0%). In the 35–44 year age group teachers were the most 25 (50.0%), followed by civil servants 17 (34.0%) others 4 (8.0%) and those engaged in skilled occupation 3 (6.0%) (Table 1).

**Table 1:** The socio-demographic characteristics of individuals examined for oral conditions

Characteristics	Age group (years)			
	n = 50 5–6 years	n = 50 12 years	n = 50 35–44 years	N = 150 Total
	n (%)	n (%)	n (%)	n (%)
Sex				
Male	28 (56.0)	26 (52.0)	20 (40.0)	74 (49.0)
Female	22 (44.0)	24 (48.0)	30 (60.0)	76 (51.0)
Father’s/individuals occupation				
Unskilled	4 (8.0)	3 (6.0)	1 (2.0)	8 (5.3)
Skilled	35 (70.0)	19 (38.0)	3 (6.0)	57 (38.0)
Teacher	3 (6.0)	10 (20.0)	25 (50.0)	38 (25.3)
Civil servant	4 (8.0)	8 (16.0)	17 (34.0)	29 (19.3)
Others	4 (8.0)	10 (20.0)	4 (8.0)	18 (12.0)

### Prevalence of oral conditions

The most prevalent oral conditions were periodontal related symptoms like plaque which was found in 100 subjects (66.0%), surface calculus in 99 (66.0%) and gingivitis in 45 (30.0%) individuals while decayed teeth were found in 20 (13.0%). There was a significant difference across the different age groups for the presence of plaque, calculus, gingivitis and enamel wear respectively with a p value of < 0.05. The 12 year age group had the most cases of plaque, calculus and gingivitis, followed by the 5–6 year olds then the 35–44 year olds. However for enamel wear the most cases were seen among the 35–44 year olds (Table 2).

**Table 2:** Frequency distribution of oral conditions by age groups

Oral condition	n = 50 5–6 years (%)	n = 50 12 years (%)	n = 50 35–44 years	N = 150 Total (%)	p value
Plaque	39 (78.0)	43 (86.0)	18 (36.0)	100 (66.0)	0.0001
Calculus	34 (68.0)	43 (86.0)	22 (44.0)	99 (66.0)	0.0001
Gingivitis	11 (22.0)	23 (46.0)	11 (22.0)	45 (30.0)	0.0100
Enamel wear	6 (12.0)	4 (8.0)	13 (26.0)	23 (15.0)	0.0320
Decayed teeth	7 (14.0)	2 (4.0)	11 (22.0)	20 (13.0)	0.2690
Trauma	2 (4.0)	2 (4.0)	4 (6.0)	8 (5.0)	Not valid
Periodontitis	0 (0.0)	0 (0.0)	3 (6.0)	3 (2.0)	Not valid
Unclassified swelling	0 (0.0)	0 (0.0)	2 (4.0)	2 (1.0)	Not valid

### Oral conditions in different occupations

The association between oral conditions and the occupation of the father or individual was compared. The occupations considered were unskilled, skilled, teacher, civil servant and others. The term skilled in this study was used to refer to individuals whose occupation includes masons, carpenters and so on while the term unskilled was for petty traders and the like. There was no significant difference between having gingivitis, periodontitis and bleeding gums and the different occupations (p > 0.05). The results for plaque showed that 49 (49.0%) of those engaged in skilled occupation had plaque and 18 (18.0%) of the teachers. For calculus 45 (45.5%) of the skilled had calculus and 19 (19.2%) of the teachers. The proportion of respondents with plaque and calculus were significantly different among the different occupations (p < 0.001) (Table 3). Also if one looks at the different occupations in Table 3 it was observed that more than 50% of those examined in each occupation group had plaque and calculus. For example 49 out of 57 (85%) skilled had plaque and 8 out 8 (100%) unskilled had calculus.

**Table 3:** Distribution of plaque and calculus based on occupation of individual or parent

Occupation	Plaque			Calculus		
	Present n (%)	Absent n (%)	Total N (%)	Present n (%)	Absent n (%)	Total N (%)
Skilled	49 (49.0)	8 (16.0)	57 (38.0)	45 (45.5)	12 (23.5)	57 (38.0)
Teacher	18 (18.0)	20 (40.0)	38 (25.3)	19 (19.2)	19 (37.3)	38 (25.3)
Civil servant	17 (17.0)	12 (24.0)	29 (19.3)	17 (17.2)	12 (23.5)	29 (19.3)
Others	10 (10.0)	8 (16.0)	18 (12.0)	10 (10.1)	8 (15.7)	18 (12.0)
Unskilled	6 (6.0)	2 (4.0)	8 (5.3)	8 (8.1)	0 (0.0)	8 (5.3)
Total	100 (66.7)	50 (33.3)	150 (100)	99 (66)	51 (34)	150 (100)
	(χ = 18.014, df = 4, p = 0.001)			(χ = 14.29, df = 4, p = 0.006)		

The relationship between father’s/individual’s occupation and oral conditions when checked for in the age groups 5–6 and 12 years had the greatest proportion that had plaque and calculus among the skilled also (Table 4).

**Table 4:** Distribution of plaque and calculus in different occupations/father’s occupation by age groups

Age	Occupation	Plaque			Calculus		
		Present n (%)	Absent n (%)	Total (50) N (%)	Present n (%)	Absent n (%)	Total (50) N (%)
5–6 years	Unskilled	4 (10.3)	0 (0.0)	4 (8.0)	4 (11.8)	0 (0.0)	4 (8.0)
	Skilled	31 (79.5)	4 (36.4)	35 (70.0)	27 (79.4)	8 (50.0)	35 (70.0)
	Teacher	0 (0.0)	3 (27.3)	3 (6.0)	1 (2.9)	2 (12.5)	3 (6.0)
	Civil servant	2 (5.1)	2 (18.2)	4 (8.0)	0 (0)	4 (25.0)	4 (8.0)
	Others	2 (5.1)	2 (18.2)	4 (8.0)	2 (5.9)	2 (12.5)	4 (8.0)
12 years	Unskilled	1 (2.3)	2 (28.6)	3 (6.0)	3 (7.0)	0 (0.0)	3 (6.0)
	Skilled	17 (39.5)	2 (28.6)	19 (38.0)	17 (39.5)	2 (50.0)	19 (38.0)
	Teacher	9 (20.9)	1 (14.3)	10 (20.0)	8 (18.6)	2 (12.5)	10 (20.0)
	Civil servant	8 (18.6)	0 (0.0)	8 (16.0)	8 (18.6)	0 (0.0)	8 (16.0)
	Others	8 (18.6)	2 (28.6)	10 (20.0)	7 (16.3)	3 (12.5)	10 (20.0)
35–44 years	Unskilled	1 (5.6)	0 (0.0)	1 (2.0)	1 (4.5)	0 (0.0)	4 (8.0)
	Skilled	1 (5.6)	2 (6.3)	3 (6.0)	1 (4.5)	2 (7.1)	3 (6.0)
	Teacher	9 (50.0)	16 (50.0)	25 (50.0)	10 (45.5)	15 (53.6	25 (50.0)
	Civil servant	7 (38.9)	10 (31.3)	17 (34.0)	9 (40.9)	8 (28.6)	17 (34.0)
	Others	0 (0.0)	4 (12.5)	4 (8.0)	1 (4.5)	3 (10.7)	4 (8.0)

### Prevalence of oral conditions according to gender

There was significant difference between the males and females and the presence of gingivitis and calculus (Table 5). More of the males (77.0%) had calculus compared to the females (55.3%). For gingivitis, 39.2% of the males compared to 21.1% females had gingivitis. There was no significant difference between males and females for the following conditions plaque, bleeding gums, enamel wear, trauma, periodontitis and DMFT index (p > 0.05) (Table 5).

**Table 5:** Association between gender and prevalence of selected oral conditions

Oral conditions	Male n = 74	Female n = 76	Total N = 150	χ^2^ (p value)
	n (%)	n (%)	N (%)	
Calculus				
Present	57 (77.0)	42 (55.3)	99 (66.0)	7.91 (0.005)
Absent	17 (23.0)	34 (44.7)	51 (34.0)	
Gingivitis				
Present	29 (39.2)	16 (21.1)	45 (30)	5.87 (0.015)
Absent	45 (60.8)	60 (78.9)	105 (70)	
Plaque				
Present	54 (73.0)	46 (60.5)	100 (66.7)	2.61 (0.106)
Absent	20 (27.0)	30 (39.5)	50 (33.3)	

### Rural/urban distribution of oral conditions

There was no significant association between location and presence of an oral condition for all the oral conditions except trauma which had a p value of 0.029, with the urban area having 7 (87.5%) out of the 8 cases. There was no difference between the rural and urban areas for conditions of unclassified swelling but the 2 cases seen were in the rural areas. Difference in occurrence of enamel wear by location was also not significant but there was a higher number of people 15 (65.2%) with the condition in the urban than the rural areas 8 (34.8%).

### DMFT index of community members

Table 6 shows the DMFT scores. Twenty individuals were found to have a history of dental decay and this gave a prevalence of 13.0%. The 5–6 years group had four people with a score of 1 and two people had DMFT of 4 and one person a score of 3, 12 years group had two people with a score of 1, 35–44 years group had seven people with a score of 1 and four people with a score of 2, 3, 4 and 7, respectively. The mean DMFT index was 0.26. The mean DMFT index for 5–6 year olds was 0.3, 12 year olds 0.04 and 35–44 year olds 0.46. The DMFT index did not show any significant difference between the rural and urban areas, however in the urban areas 13 (65.0%) individuals had a DMFT score compared to 7 (35.0%) individuals in the rural areas.

**Table 6:** DMFT index of community members

Age group years	Population	D+M+F	D+M+F	DMFT index
	n	n	%	
5–6	50	15	37.5	0.30
12	50	2	5.0	0.04
35–44	50	23	57.5	0.46
Total	150	40	100.0	0.26

## Discussion

### Prevalence of oral conditions

Plaque and calculus were the most common conditions seen in all the age groups in this study. This is not surprising since plaque is one of the most prevalent oral conditions and is found in the mouth of most human beings at one stage or the other [15]. Socioeconomic status has been known to influence the presence or absence of oral conditions. In this study we find that most of the pupils and students came from government or public schools which usually have children of the lower socioeconomic class. Some other studies have shown a greater number of individuals with plaque and calculus among students in public rather than private schools [16]. There were no elite or private schools in the sample size and this may explain why there were more cases of plaque and calculus than caries. This is because previous studies in Nigeria have shown that caries is more in children attending private schools than government schools because they are more financially empowered to buy cariogenic foods [17].

The relationship between occupation and oral conditions was checked in the various age groups. Among the 5–6 and 12 year olds most of those with plaque and calculus were those whose fathers were among the skilled and unskilled workforce. In this study 79.5% of the 5–6 years old that had plaque and calculus were those whose fathers were among the skilled labour force and for the 12 year olds it was 39.5%. While the only two individuals with bleeding gums among 5–6 years age group and the only one individual with bleeding gums among the 12 year olds had parents who were among the skilled workforce. Lower socioeconomic status has been associated with more periodontal disease because it is directly related to oral cleanliness which is inversely related to periodontal conditions [16]. In this study these children had more plaque and calculus which is usually a reflection of the state of oral cleanliness. From this study at least half of the individuals in all the occupations have plaque and calculus showing the wide prevalence (Table 3). In this study the prevalence of gingivitis was lower than plaque and calculus among adults and children. The prevalence of gingivitis was 30.0% across all the age groups while the prevalence of gingivitis among those in age group 5–6 and 35–44 years was 22.0% while for 12 years it was 46.0%. Taiwo in 1993 reported 27.0% in Nigerian children below 12 years old in a dental clinic located in Western Nigeria and 67.0% in children between 2–3 years old with poor oral hygiene [18]. In Burkina Faso, consistent with community periodontal index data reported for African children and adults of 6, 12, 18 and 35–45 years they had high levels of gingival bleeding and calculus attributable to poor oral hygiene practices [19]. It is well documented that most studies indicate a high prevalence of periodontal disease in Nigerians [20]. Therefore although lower socioeconomic class has shown greater levels of periodontal conditions in this study there is a concern that periodontal diseases still affect a wide proportion of the population. This brings to attention the need for greater oral health awareness and health education. Information obtained from this study will help in the planning and provision of oral health services and to provide appropriate oral health education.

When gender was considered there were significantly more males with calculus than females in this study. It is not surprising that males have more calculus than females since women are known to have better oral hygiene practices [21]. In a national pathfinder study conducted in Nigeria it was shown that the percentage of subjects with good oral health was very small in all age groups while the majority of subjects had calculus [22]. A survey of the oral health status of children and adults in Burkina Faso showed calculus was dominant for all ages [19]. The results obtained in this study are consistent with other studies in different parts of the world. The progression from having plaque or calculus to periodontal diseases like gingivitis and periodontitis is expected with time and especially among the older age since there is a correlation between oral hygiene status and presence of periodontal diseases [6]. Although in this study the prevalence of gingivitis was low and periodontitis even lower across all the age groups, the 3 cases of periodontitis found in this study were among the 35–44 years age group which is to be expected since it occurs among older ages. The proportion that had calculus was 44% and for plaque 36% among the age group 35–44 year olds; if these conditions are not treated it is likely that these individuals will develop any of these oral diseases like periodontitis in future. This corresponds to what was seen in other studies where these diseases are found in older age groups [6].

It also shows the importance of school dental health and community health programmes where trained teachers, mothers, and health workers can play a role in promotion of oral health issues among various groups.

### Other conditions

Although it is known that dental caries and periodontal diseases are the most common oral diseases worldwide, other conditions are known to occur in Africa such as acute necrotizing ulcerative gingivitis (ANUG), oral manifestations of HIV, maxillofacial trauma and benign tumours as well as certain harmful practices like trimming of teeth and forceful extraction of teeth germs [23]. This study may not be able to detect some of these other conditions whose prevalence may be affected by sample size, location, ethnicity and other factors.

However other conditions found in this study like enamel wear had a prevalence of 15.0%, while conditions like trauma and bleeding gums had a prevalence of < 6.0%. Across all age groups present in this study 4.0% had bleeding gums, while only one person among the 12 year olds had gingival recession. Periodontitis was absent among the 5–6 and 12 year olds. However among the 35–44 year olds 6.0% had gingival recession and periodontitis.

The caries experience for this population was low with overall 13.0% prevalence. In the age groups it was higher among the adults 35–44 years (22.0%), then the 5–6 years old (14.0%) and 12 year olds (4.0%). The mean DMFT index was 0.26 for all age groups in this study, while among 5–6 years it was 0.30, 12 years 0.04 and 35–44 years 0.46. Dental caries is known to be more common among those who are exposed to certain risk factors like refined carbohydrate diet which is more common in urban areas and among the elite. In this study most of the participants were from government schools and not private schools. Similarly the adults were teachers in those schools or workers in the local government secretariat. The elite were not part of the sample population and this could contribute to the low caries prevalence. However this may not be the only reason since the role of socioeconomic factors in caries prevalence may be influenced by other factors in populations where sweets and refined carbohydrates are readily available and affordable to all. The greater prevalence of caries in the 35–44 years group may be attributed to the fact that caries experience is known to increase with age. It was also seen in the Umesi et al. study on dental caries that older adolescents had more dental caries [5].

Also in this study the cases of caries seen were more in the urban areas than the rural especially the 35–44 age group. Urban areas usually have more cases of caries than rural areas due to greater availability of refined diet. In comparison nearly half (46.0%) of the children (8 days–16 years) in the University of Port-Harcourt Teaching Hospital (UPTH) clinic had dental caries [24]. This may be attributed to the higher socioeconomic class of children who patronize UPTH.

The caries prevalence was a bit higher among the females than the males in this study though not significantly different. In a national oral health survey in China among adults, caries experience was higher in females than in males [25]. In Burkina Faso among the adults dental caries prevalence was higher for women than men [19]. Most studies show that females have more cases of dental caries than males. This may be attributed to the dietary habits and earlier pattern of tooth eruption among females [24].

There was no significant difference between rural and urban locations for almost all the oral conditions found in the study. Trauma was the only significant condition with more cases of trauma found within the urban area. This is to be expected since urban areas are known to be prone to accidents due to more vehicles, traffic and people. There was no difference between the rural and urban areas for conditions of unclassified swelling but the 2 cases seen were in the rural areas. Such individuals with the swellings may not have been able to access the necessary care because of lack of trained staff on oral health at the health facilities. Enamel wear too was not significant, but there was a higher number of people with the condition in the urban areas than the rural. The reason for this is not clear since coarse and fibrous diets which lead to enamel wear are more common in rural areas. In this study it was noticed that even the 5–6 year olds had cases of enamel wear and all were in the urban areas while for other age groups it was almost equal in urban and rural. Tooth wear lesions can be as a result of chemical erosion; and consumption of acidic drinks is more in urban areas. So this finding may be the result of a shift in diet in urban areas but more research is required to verify this.

## Conclusion

Oral health in communities of Kwara State is sub optimal. The presence of oral conditions is influenced by age, occupation, location and gender. The most common oral conditions plaque and calculus are related to poor oral hygiene. Oral health services need to be intensified and made accessible in urban and rural areas. Intervention targeting different age groups and the most common oral lesions is suggested. Demographic factors which influenced the prevalence of some oral conditions can also be considered in planning intervention strategies.

## Limitations

One of the limitations of this study was that it was part of a larger research that involved other study populations and there was a time constraint which necessitated sticking to the minimum sample size in each LGA. It would have probably been more robust with a larger sample size and there is possibility some more of the results in the age groups may have been statistically significant.

The blinding of the investigators to the location as to whether urban or rural would have further enhanced the validity. The fact that the number of years one resided in the particular urban or rural location was not considered may influence the results obtained and give wrong assumptions on oral conditions present in either locations.

## Additional file

**Additional file 1.** Oral health conditions for abstract. The data contains the socio-demographic characteristics and oral health conditions found among the 150 participants.

## Abbreviations

WHO: World Health Organization
DMFT: decayed missing filled teeth
HIV: human immunodeficiency virus
LGA: local government area
UBE: universal basic education
SPSS: statistical package for social science
CP2: community periodontal index
ANUG: acute necrotizing ulcerative gingivitis
UPTH: University of Port Harcourt Teaching Hospital
UITH: University of Ilorin Teaching Hospital
